# Hemolytic anemia following intravenous immunoglobulin therapy in patients treated for Kawasaki disease: a report of 4 cases

**DOI:** 10.1186/1546-0096-10-10

**Published:** 2012-04-16

**Authors:** Roberta Berard, Blair Whittemore, Rosie Scuccimarri

**Affiliations:** 1 Department of Pediatrics, Section of Rheumatology, Children's Hospital, London Health Sciences Centre, 800 Commissioners Road East, London, Ontario N6A 5W9, Canada; 2 Department of Pediatrics, Division of Hematology/Oncology, Montreal Children's Hospital, McGill University Health Centre, 2300 Tupper Street, Montreal, Quebec H3H 1P3, Canada; 3 Department of Pediatrics, Division of Rheumatology, Montreal Children's Hospital, McGill University Health Centre, 2300 Tupper Street, Montreal, Quebec H3H 1P3, Canada

**Keywords:** Direct antiglobulin test, Isohemagglutinins, Retreatment

## Abstract

**Background:**

Hemolytic anemia is a rare but reported side effect of intravenous immunoglobulin (IVIG) therapy. The risk of significant hemolysis appears greater in those patients who receive high dose IVIG. The etiology is multifactorial but may relate to the quantity of blood group antibodies administered via the IVIG product.

**Findings:**

We describe 4 patients with significant hemolytic anemia following treatment with IVIG for Kawasaki disease (KD). Direct antibody mediated attack as one of the mechanisms for hemolysis, in this population, is supported by the demonstration of specific blood group antibodies in addition to a positive direct antiglobulin test in our patients.

**Conclusions:**

Clinicians should be aware of this complication and hemoglobin should be closely monitored following high dose IVIG therapy.

## Background

KD is a systemic vasculitis in which the major complication is the development of coronary artery aneurysms (CAA). Treatment with intravenous immunoglobulin (IVIG) significantly lowers the incidence of CAA. Standard therapy for the treatment of KD is high dose IVIG (2 g/kg) and aspirin. Retreatment with IVIG is administered for persistence of fever ≥ 36 h after the first infusion. Persistence of fever after initial IVIG therapy is estimated to occur in approximately 10% - 20% of cases [1].

IVIG is used in high doses, most frequently at 2 g/kg, as an immunomodulatory agent [2]. It is a pooled blood product acquired from thousands of blood donors and it contains measurable levels of anti-A and anti-B (IgG subclass) as well as non-ABO erythrocyte antibodies (e.g. anti-D) [3]. IVIG is considered to be a safe product that is generally well tolerated. Hemolysis is a rarely reported side effect of IVIG. It occurs more commonly in those patients who receive high-dose IVIG [2,4] as is used in the treatment of KD. In the literature, there are 6 reported cases of children with hemolytic anemia following IVIG treatment for KD [4-7]. In this report, we describe 4 patients, all from a single centre, who developed hemolytic anemia following IVIG treatment for KD. To our knowledge, this is one of the largest case series describing this complication in this patient population.

## Findings

Significant hemolysis was noted in 4 out of 25 (16%) patients diagnosed and treated for KD at our centre during a 14-month interval. In this cohort of 25 patients, 9 (36%) required retreatment with IVIG for persistent fever. This was higher than our usual retreatment rate of 18% [8]. Of these 9 patients, 4 (44.4%) developed significant hemolytic anemia and of these, 2 required blood transfusion for hemodynamic instability. In all 4 patients, the direct antiglobulin test (IgG) was positive. In the 3 patients tested, all demonstrated specific blood group antibodies in the eluates prepared from their red cells (see Table 1).

## Case presentations

### Case 1

A previously healthy 22-month old Egyptian male presented with 13 days of fever, diffuse maculopapular rash, conjunctival injection without exudate, erythema and edema of the hands and feet and oral mucosal changes. The patient was diagnosed with KD and treated with IVIG. Approximately 36 h after completion of the 1^st ^IVIG, he was given a 2^nd ^infusion of IVIG because of persistent fever. Significant hemolytic anemia was noted 30 h after completion of the 2^nd ^IVIG (see Tables 1 and 2). The patient was also treated with oral prednisone (1 mg/kg) that was tapered over 6-weeks. Two weeks following the diagnosis of KD, a small aneurysm of the left anterior descending coronary artery (3.9 mm) was noted and the child was continued on aspirin therapy.

### Case 2

A previously healthy 4 year old Caucasian girl presented with 9 days of fever, diffuse maculopapular rash, conjunctival injection without exudate, erythema of the hands and feet with periungual desquamation, and oral mucosal changes. The patient was diagnosed with KD and treated with IVIG. Approximately, 50 h after completion of the 1^st ^IVIG, the patient was given a 2^nd ^infusion of IVIG for recrudescence of fever. Prior to the 2^nd ^dose of IVIG, she was evaluated for hemolytic anemia because of a drop in hemoglobin (see Table 2). At that time, she had a normal LDH and bilirubin, as well as a negative direct antiglobulin test. Unfortunately, significant hemolytic anemia occurred following the 2^nd ^dose of IVIG. This was noted 48 h following the completion of the 2^nd ^IVIG (see Table 1). The patient made a good clinical recovery without cardiovascular complications of KD.

### Case 3

A previously healthy 16-year old Asian male presented with 8 days of fever, diffuse maculopapular rash, conjunctival injection without exudate, erythema and edema of the feet, and oral mucosal changes. The clinical course was complicated by myocarditis, requiring intensive care unit admission and inotropic support, cholestatic hepatitis and pancreatitis. Extensive infectious disease work-up was negative. The patient was diagnosed with KD. Given his atypical presentation, the diagnosis was made only after an extensive work-up excluding other possible diagnoses. He was treated with IVIG. A 2^nd ^infusion of IVIG was given 72 h later for recrudescence of fever. Towards the end of the 2^nd ^IVIG infusion, the patient developed "tea-colored" urine. Hemolytic anemia was suspected and confirmed (see Tables 1 and 2). A few days later, he required a red cell transfusion for hemodynamic instability secondary to hemolytic anemia. Intravenous pulse methylprednisolone (30 mg/kg) was also given for persistent fever. On follow-up, he developed typical periungual desquamation associated with KD. He made a good clinical recovery without cardiovascular complications.

### Case 4

A previously healthy 7-month old Caucasian boy presented with 5 days of fever, diffuse maculopapular rash, conjunctival injection without exudate, erythema of the hands and feet and oral mucosal changes. The patient was diagnosed with KD and treated with IVIG. Approximately 42 h after completion of the first IVIG, he was given a 2^nd ^infusion of IVIG for persistence of fever. Patient was noted to be very pale and lethargic approximately 8 h after the 2^nd ^IVIG was completed. Significant hemolytic anemia was confirmed (see Tables 1 and 2). Due to hemodynamic instability and difficulty with vascular access, he required intraoperative placement of an intravenous line for receipt of a red cell transfusion. Two weeks following the diagnosis of KD, a small right coronary artery aneurysm (3.0 mm) was noted that resolved after 2 months.

**Table 1:** Clinical characteristics and laboratory investigation in KD patients with hemolytic anemia following IVIG

	Case 1	Case 2	Case 3	Case 4
**Gender**	Male	Female	Male	Male

**Age at diagnosis**	22 months	4 years	16 years	7 months

**Duration of fever before treatment**	13 days	9 days	8 days	5 days

**Clinical criteria of KD**	4/5	4/5	4/5	4/5

**Hemoglobin (g/L) before 1^st ^IVIG**	94 (115-135)	117 (115-155)	114 (130-160)	93 (105-135)

**Lowest hemoglobin (g/L) after 2^nd ^IVIG**	65	64	56	56

**Blood group**	A Rh(D) pos	A Rh(D) pos	B Rh(D) pos	AB Rh(D) pos

**Direct antiglobulin test baseline (IgG)**	ND*	Negative	Negative	ND*

**Direct antiglobulin test after hemolysis (IgG)**	Positive	Positive	Positive	Positive

**Antibody identified in red blood cell eluate**	Anti-A	ND*	Anti-B	Anti-A, Anti-B

**LDH (U/L)**	660 (470-920)	522 (142-297)	1881 (340-750)	495 (140-304)

**Indirect bilirubin (umol/L)**	12 (0-34)	20 (0-34)	55 (0-19)	29 (0-34)

**Haptoglobin (0.69 - 1.96 g/L)**	3.44	ND*	0.06	0.38

**Significant spherocytosis**	Yes	Yes	Yes	Yes

**Reticulocyte count (0.2 - 2%)**	13.1	12.2	4.7	2.4

**Blood transfusion**	No	No	Yes	Yes

**Coronary outcome**	CAA	Normal	Normal	CAA

ND* Not Done; *CAA *coronary artery aneurysm

**Table 2:** Hemoglobin in KD patients with hemolytic anemia following IVIG

	Case 1	Case 2	Case 3	Case 4
**Hemoglobin (g/L)** **Before 1^st ^IVIG**	94 (115-135)	117 (115-155)	114 (130-160)	93 (105-135)

**Hemoglobin (g/L)** **Before 2^nd ^IVIG**	ND	91*	102	86

**Hemoglobin (g/L)** **24**-**48 hours after 2^nd ^IVIG**	65	74 (64 - at 72 h)	56	56

*ND *Not Done; *Suspected hemolysis prior to 2^nd ^IVIG however, work-up was negative

## Discussion

Hemolytic anemia is a potentially serious and possibly under recognized side effect of IVIG therapy. The presence of transient, passively acquired antibodies (positive direct antiglobulin test) with associated decreased haptoglobin levels and mild reticulocytosis following treatment with IVIG was noted many years ago [9]. However, this rarely results in clinically significant anemia. There have only been 4 previous reports with a total of 6 patients described to have hemolysis following IVIG therapy for KD [4-7]. In all 4 reports, the presumed mechanism was of a direct antibody mediated attack, but they only demonstrated specific blood group antibodies in 4 of 6 of these patients [4-7] We found antibodies in the eluate of the 3 patients that were tested; in addition, all 4 patients had a positive direct antiglobulin test (see Table 1). To our knowledge this is the largest case series that demonstrated specific blood group antibodies following IVIG for Kawasaki disease, which further supports the hypothesis that direct antibody mediated attack is one of the potential mechanisms for hemolysis in this population.

The etiology of clinically significant hemolysis in patients with KD treated with IVIG is multifactorial. Isohemagglutinins (anti-A/B antibodies) present in IVIG can cause direct antibody attack on RBCs. Even though the titers of these antibodies, in commercially available products, must meet agreed-upon specifications by manufacturers through a Biologics Licence Application [3], there are many documented cases of hemolytic anemia despite these limits [10]. This phenomenon may be dose dependent given that in 6 of the 6 patients previously reported, and in all 4 of our patients (total 10/10 patients), acute hemolytic anemia occurred following a second dose of IVIG for persistent fever. All patients received a total of 4 g/kg prior to the clinically significant hemolysis. However, it is known that hemolytic anemia can occur even with doses as low as 1 g/kg [10]. Factors that may increase the risk for hemolysis include the dose of IVIG, the titer of the isohemagglutinin antibody in the IVIG preparation, the strength of the patient's antigen expression [5,7], the affinity of the antibody for the antigen [7], or a combination of these factors.

All our KD patients received Gammunex. Hemolytic anemia in KD has also been reported with Gammaguard [7]. Isosmolar, liquid IVIG products, such as Gammaguard and Gammunex, seem to have higher isohemagglutinin titers than lyophilized products [10]. However, lyophilized products are more osmolar and have other potential side effects including acute renal failure and thrombosis [10].

There is a greater risk for hemolysis in patients with non-O blood groups [10]. It is hypothesized that patients with non-O blood groups, with low concentrations (congenital or acquired) of soluble A and/or B substance in their plasma, are at increased risk for hemolysis due to their inability to neutralize the anti-A and/or anti-B isohemagglutinins present in the plasma after IVIG infusion [11]. What is interesting about this hypothesis is that in all KD patients who developed hemolysis following IVIG (including our patients), the blood type in 6 was A+, 2 AB+, 1 B + and 1 O+. The patient who was O + also had cold agglutinins.

It is also plausible that patients with a baseline anemia, as is often seen in KD, may be more susceptible to hemolysis because of an absolute decrease in binding sites to disperse the antibodies. Other potential causes for hemolysis in KD include cold agglutinating anti-RBC antibodies [4]. KD in itself may also be a predisposing factor for hemolysis given that there is immune dysregulation in this disease. This is supported by case reports describing hemolysis in patients with KD who did not receive IVIG [12,13].

Another possible mechanism for a decrease in hemoglobin with IVIG, independent of isoantibodies, is enhanced erythrocyte sequestration [14]. Since IVIG contains high molecular weight IgG complexes, these can mimic immune complexes by activating complement [14]. These complexes bind to complement receptor 1 on RBCs which leads to erythrophagocytosis and hence a reduction in hemoglobin [14]. Predictors for this phenomenon include age and RBC ability to bind the IVIG immune complex-like moieties [14].

In Canada, the prevalence of hemolysis following IVIG is unknown given the underdeveloped surveillance system of IVIG administration. However, in recent years, the Canadian Blood Services and Hema-Quebec have noted an increase in reporting of this complication [15].

We witnessed an unusually high rate (16%) of clinically significant hemolysis over a 14-month period at our institution and this complication had not been noted previously. We are unaware of any important changes that occurred in the IVIG product during this time period to account for these findings. At the same time, we experienced a high retreatment rate and therefore we cannot exclude subtle changes in the IVIG product that could explain both these findings. Specific blood group antibodies were demonstrated in addition to the positive direct antiglobulin test, which suggests that the primary mechanism for hemolysis in our cases was through direct antibody mediated attack.

## Conclusions

Hemolytic anemia should be considered in patients with KD treated with high dose IVIG who experience a drop in hemoglobin following treatment and/or who develop clinical signs of hemolysis. It is important that physicians are aware of this potential complication for early recognition as well as for disclosing this potential side effect to patients and their families. It is now our routine practice to monitor the hemoglobin 24 to 48 h after completion of IVIG and one week after discharge, particularly if retreatment is necessary. A work up for hemolysis is not routinely required except if this complication is suspected. Families should be informed to return should their child develop pallor, lethargy, dark urine, shortness of breath or palpitations. Further insight is still required in regards to the pathogenesis and predisposing factors for hemolysis in this patient population.

## Consent

A waiver for case reports was approved from the Director of Professional Services, Montreal Children's Hospital, McGill University.

## Competing interests

The authors declare that they have no competing interests.

## Authors' contributions

RB - reviewed medical records, gathered patient data, literature review, manuscript preparation; BW - interpretation of hematology tests, manuscript review; RS - reviewed medical records, literature review, manuscript preparation. All authors read and approved the final manuscript.
